# The type of gastrectomy and modified frailty index as useful predictive indicators for 1-year readmission due to nutritional difficulty in patients who undergo gastrectomy for gastric cancer

**DOI:** 10.1186/s12893-021-01450-6

**Published:** 2021-12-29

**Authors:** Tomohiro Osaki, Hiroaki Saito, Wataru Miyauchi, Yuji Shishido, Kozo Miyatani, Tomoyuki Matsunaga, Shigeru Tatebe, Yoshiyuki Fujiwara

**Affiliations:** 1 Department of Surgery, Tottori Prefectural Central Hospital, 730 Ezu, Tottori, 680-0901 Japan; 2 Department of Surgery, Japanese Red Cross Tottori Hospital, 117 Shotoku-cho, Tottori, 680-8571 Japan; 3 Division of Gastrointestinal and Pediatric Surgery, Department of Surgery, School of Medicine, Faculty of Medicine, Tottori University, 36-1 Nishi-cho, Yonago, 683-8504 Japan

**Keywords:** Gastric cancer, Gastrectomy, Modified frailty index, Nutritional difficulty, Readmission

## Abstract

**Background:**

Patients who undergo gastrectomy for gastric cancer (GC) are likely to have nutritional difficulty after surgery. Readmission due to nutritional difficulty is common in such patients. Thus, in this study, we aim to identify the predictive indicators for readmission due to nutritional difficulty in patients who underwent gastrectomy for GC.

**Methods:**

We retrospectively reviewed surgical outcomes in 516 consecutive patients who underwent gastrectomy for GC.

**Results:**

The readmission rate within 1 year was 13.8%. Readmission due to nutritional difficulty was observed in 20 patients (3.9%); it was determined as the second leading cause of readmission. Multivariate analysis revealed that the type of gastrectomy and the modified frailty index (mFI) were independent predictive indicators of readmission due to nutritional difficulty. Patients were assigned 1 point for each predictive indicator, and the total points were calculated (point 0, point 1, or point 2). The readmission rates due to nutritional difficulty were 1.2%, 4.7%, and 11.5% in patients with 0, 1, and 2 points, respectively (*P* = 0.0008).

**Conclusions:**

The readmission rate due to nutritional difficulty was noted to be high in patients who underwent total or proximal partial gastrectomy with high mFI. Intensive follow-up and nutritional support are needed to reduce readmissions due to nutritional difficulty. Reduced readmission rates can improve patient quality of life and reduce medical costs.

## Background

Hospital readmissions can only result in increasing medical costs. According to Stephen et al., 19.6% of Medicare patients were rehospitalized within 30 days [1], resulting in an estimated excess healthcare cost of 17.4 billion USD. Thus, the Affordable Care Act mandated the establishment of the Hospital Readmissions Reduction Program, which penalized payments to hospitals with excess readmissions. In addition to increased medical costs, readmission can worsen patient quality of life (QOL). Readmission often leads to prolonged hospitalization, preventing patients from returning to their regular lives. Furthermore, readmission is significantly associated with poor prognosis [2, 3]. Therefore, avoiding readmission after surgery is deemed very important. To this end, the development of reliable predictors of readmission after surgery is of significance.

Gastric cancer (GC) has been identified as one of the most common malignancies worldwide [4]. Gastrectomy with regional lymph node dissection is the mainstay of curative treatment for GC. Gastrectomy is associated with poor food intake due to decreased stomach volume. Thus, hospital readmissions due to nutritional difficulty are common in patients who have undergone gastrectomy. A meta-analysis demonstrated an 8% incidence of 30-day readmissions after radical gastrectomy (range, 4–12%) [5]. The main causes for the 30-day readmissions were nutritional difficulty and surgical site infections. Nutritional difficulty was also one of the main causes of readmission in patients who underwent gastrectomy for GC. Planned nutritional support improved the nutritional status of patients who underwent gastrectomy for GC [6, 7]. Therefore, readmission due to nutritional difficulty may be avoided by intensive nutritional support. If predictive indicators of nutritional difficulty are identified, patients who are at high risk of readmission due to nutritional difficulty after gastrectomy can be selected for more intensive treatment and observation. However, such indicators are yet to be identified in patients with GC. Thus, in this current study, we aimed to identify the predictive indicators for readmission due to nutritional difficulty in patients who underwent gastrectomy for GC.

## Methods

### Patients

Between January 2010 and December 2017, 516 consecutive patients with a pathological diagnosis of gastric adenocarcinoma who underwent gastrectomy at Tottori University Hospital were enrolled in this study. Patients with gastric tube cancer and synchronous primary cancer were excluded from this study. The enrolled patients underwent distal partial gastrectomy (DG), total gastrectomy (TG), or proximal partial gastrectomy (PG) with dissection of the regional lymph nodes. Patient information was obtained by retrospectively reviewing the hospital’s database. The institutional review board of Tottori University Hospital approved this study (Approval number: 17A152), and the requirement for informed consent was waived for this retrospective study. Clinicopathologic findings were based on the 15th Edition of the Japanese Classification of Gastric Carcinoma [8].

Readmission was defined as hospitalization after primary discharge due to unexpected causes associated with GC, surgery, or treatment for GC. Although admission after primary discharge for planned chemotherapy was not considered to be readmission in this study, admission due to adverse events associated with chemotherapy was considered as a readmission. We examined readmission within 1 year, as the condition of patients who have undergone gastrectomy for GC is noted to be generally unstable 1 year after surgery. In fact, Kim et al. reported that approximately 80% of readmissions were observed within 1 year after surgery in patients with GC who underwent gastrectomy [9]. When patients had multiple causes for readmission, the most significant one was recorded as the cause of readmission. Patients were classified as having nutritional difficulty if they were unable to ingest the necessary amount of nutrients due to intolerance of oral intake after the gastrectomy. In principle, we admitted patients unable to perform normal daily activities due to nutritional difficulty to the hospital and administered parenteral nutrition.

### Modified frailty index

The modified frailty index (mFI) is based on 11 physiological deficits, derived from the original 70-item Canadian Study of Health and Aging Frailty Index [10]. Patients were assigned 1 point for each of the 11 physiological deficits, and the total points assigned to each patient were divided by 11. A higher score indicated increased frailty [11].

### Prognostic nutritional index

The serum albumin concentration and total lymphocyte count in the peripheral blood were measured within 1 month before surgery. The prognostic nutritional index (PNI) was calculated using the formula as follows: 10 × serum albumin level + 0.005 × total peripheral lymphocyte count [12].

### Statistical analysis

Continuous variables were expressed as mean ± standard deviation. Differences in the interval between primary discharge and first readmission and the number of readmissions in patients readmitted due to nutritional difficulty versus patients readmitted due to other causes were determined using Mann–Whitney U-test. Differences between the categorized variables were determined using the Chi-squared test. The optimal cutoffs for continuous variables (age, body mass index, PNI, and mFI) in the readmission analysis were determined with the Youden index using a receiver operating characteristic (ROC) analysis. Univariate and multivariate analyses were then performed to identify the predictive indicators for readmission using logistic regression analysis. A stepwise procedure was used to identify possible predictive factors for readmission in the multivariate analysis. *P* < 0.05 was considered statistically significant. GraphPad Prism version 6 (GraphPad Software, Inc., La Jolla, CA, USA) and SPSS Statistics, version 24 (IBM Corp., Armonk, NY) were used for statistical analyses.

## Results

**Fig. 1:**
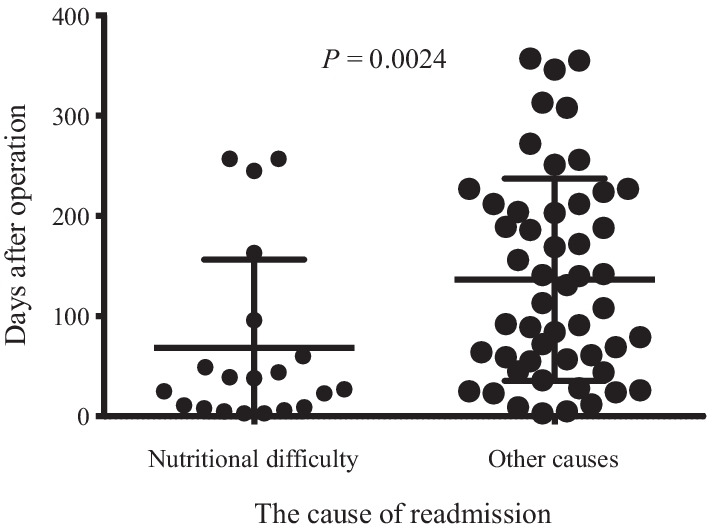
The interval from primary discharge to first readmission in patients who underwent gastrectomy for gastric cancer according to the cause of readmission

Table 1 shows the clinical features of the 516 patients included in this study; as per our findings, 94 readmissions within 1 year were observed in 71 patients (13.8%). In total, 56 patients were readmitted once (78.9%), 10 patients were readmitted twice (14.1%), 3 patients were readmitted thrice (4.2%), and 1 patient each was readmitted four and five times (1.4% each). Table 2 shows the causes of readmission. The leading cause of readmission was palliative care (n = 30), followed by nutritional difficulty (n = 25), ileus (n = 13), and chemotherapy adverse events (n = 11). Nutritional difficulty associated with chemotherapy was considered an adverse event of chemotherapy in this study. Regarding readmission for nutritional difficulty, 25 readmissions were observed in 20 patients (3.9%); 17 patients were readmitted once, 2 patients were readmitted twice, and 1 patient was readmitted four times. Figure 1 shows the intervals between primary discharge and first readmission. The 7-day, 30-day, and 90-day readmission rates were 8.5% (6/71), 26.8% (19/71), and 52.1% (37/71), respectively, across all cases. The 7-day, 30-day, and 90-day readmission rates for nutritional difficulty were 20% (4/20), 50% (10/20), and 75% (15/20), respectively. The intervals between primary discharge and first readmission due to nutritional difficulty were noted to be significantly shorter than the intervals due to other readmission causes (68.4 ± 88.1 vs. 136.4 ± 101.0 days; P = 0.0024). In contrast, no significant differences in the number of readmissions were observed when comparing patients readmitted for nutritional difficulty versus patients readmitted for other causes (1.6 ± 1.1 vs. 1.2 ± 0.6; P = 0.25).

**Table 1:** Clinical features of the patients included in this study (n = 516)

	Number of patients	(%)
Gender		
Female	139	26.9
Male	377	73.1
Age		
< 74	321	62.2
≥ 74	195	37.8
BMI (kg/m^2^)		
< 25	420	81.4
≥ 25	96	18.6
Gastrectomy		
DG	323	62.6
TG/PG	193	37.4
Surgical approach		
Laparoscopy	362	70.2
Open	154	29.8
Stage of disease		
I–III	481	93.2
IV	35	6.8
Modified frailty index		
High (≥ 0.14)	373	72.3
Low (< 0.14)	143	27.7
Solitude		
Absent	462	89.5
Present	54	10.5
PNI		
> 46.61	346	67.1
≤ 46.61	170	32.9
Complication^a^		
Absent	449	87
Present	67	13
Neoajuvant chemotherapy		
Absent	474	91.9
Present	42	8.1
Nonhome discharge		
Absent	493	95.5
Present	23	4.5
Ajuvant chemotherapy		
Absent	394	76.4
Present	122	23.6

*DG* distal partial gastrectomy, *TG* total gastrectomy, *PG* proximal partial gastrectomy

^a^Present: grade III and more according to Clavien–Dindo classification

**Table 2:** The causes of readmission and times of readmission in cumulative total 94 cases observed in 71 patients

	Number of readmission cases	%
Cause of readmission		
Palliative care	30	31.9
Nutritional difficulty	25	26.6
Ileus	13	13.8
Adverse events of chemotherapy	11	11.7
Reoperation	6	6.4
Biliary tract infection	5	5.3
Recurrence	2	2.1
Pneumonia	1	1.1
Abdominal abscess	1	1.1

	Number of patients	%
Time of readmission		
Once	56	78.9
Twice	10	14.1
Three times	3	4.2
Four times	1	1.4
Five times	1	1.4

**Fig. 2:**
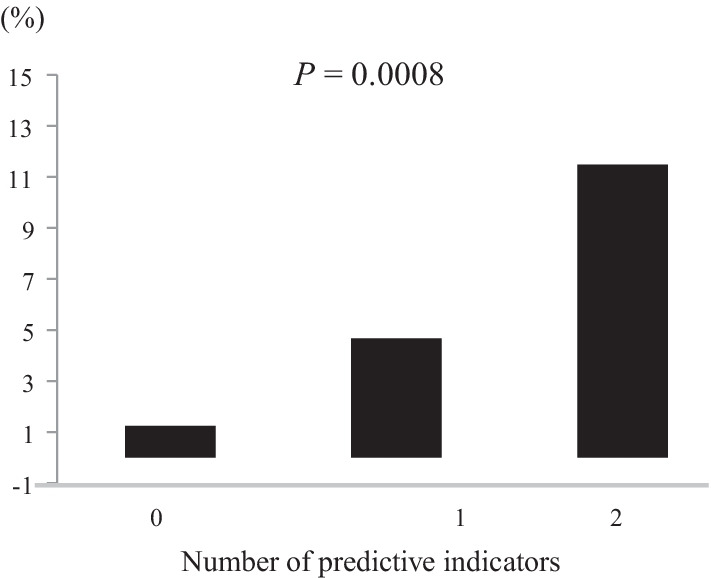
The readmission rates due to nutritional difficulty, according to the type of gastrectomy and mFI. Patients were assigned 1 point for each predictive indicator, and the total points were calculated (point 0, point 1, or point 2)

**Fig. 3:**
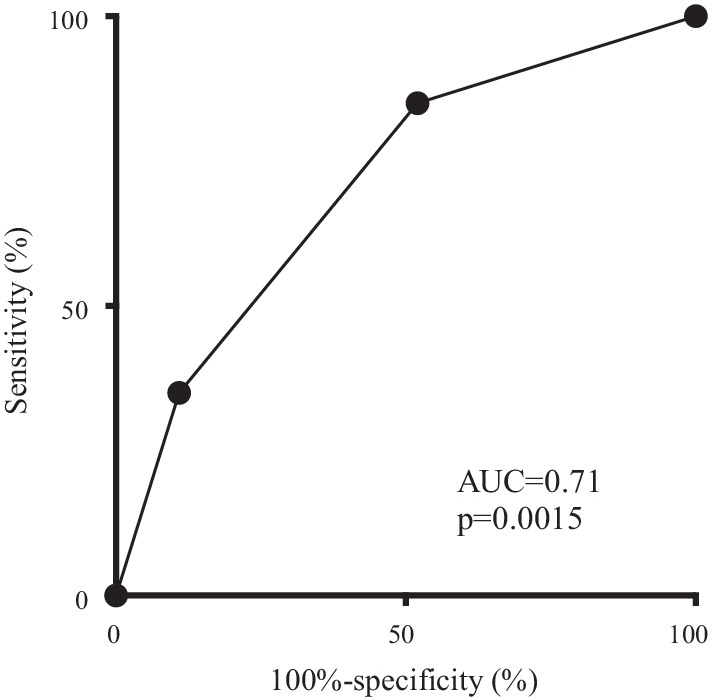
ROC curve combining the type of gastrectomy and the mFI for readmission due to nutritional difficulty

Because most readmissions due to nutritional difficulty were observed within 1 year, we determined the predictive factors of readmission within 1 year as well. Univariate analysis of the clinicopathologic characteristics revealed that age, the type of gastrectomy, mFI, and PNI were predictive indicators of readmission due to nutritional difficulty (Table 3). All parameters with differences of *P* < 0.05 in the univariate analysis were included in the multivariate analysis. The multivariate analysis and stepwise procedure revealed that mFI and the type of gastrectomy were independent predictive indicators of readmission due to nutritional difficulty (Table 3). Patients were assigned 1 point for each predictive indicator, and the total points were calculated (point 0, point 1, or point 2). The readmission rates due to nutritional difficulty were 1.2%, 4.7%, and 11.5% in patients with 0, 1, and 2 points, respectively (*P* = 0.0008; Fig. 2). Furthermore, as per our ROC analyses, it was found that the area under the curve for the number of independent predictive indicators was much higher than that of either mFI or the type of gastrectomy alone (Fig. 3).

**Table 3:** The predictive indicators for readmission due to nutritional difficulty

	Univariate analysis			Multivariate analysis		
	P	HR	95% CI	P	HR	95% CI
Gender (male vs. female)	0.230	2.14	0.62–7.42			
Age (≥ 74 vs. < 74)	0.043	2.57	1.03–6.39			
BMI (≥ 25 kg/m^2^ vs. < 25 kg/m^2^)	0.062	2.46	0.96–6.35			
Gastrectomy (TG/PG vs. DG)	0.013	3.26	1.28–8.32	0.021	3.05	1.19–7.84
Surgical approach (open vs. laparoscopy)	0.988	1.01	0.38–2.67			
Stage (IV vs. I–III)	0.150	2.56	0.71–9.19			
Modified frailty index (high vs. low)	0.008	3.37	1.37–8.32	0.014	3.15	1.27–7.83
Solitude (present vs. absent)	0.428	0.44	0.06–3.35			
PNI (≤ 46.61 vs. > 46.61)	0.012	3.21	1.29–8.01			
Complication ≥ CD3 (present vs. absent)	0.687	0.74	0.17––3.25			
Neoajuvant chemotherapy (present vs. absent)	0.757	1.27	0.28–5.66			
Nonhome discharge (present vs. absent)	0.905	1.13	0.15–8.86			
Ajuvant chemotherapy (present vs. absent)	0.497	1.40	0.53–3.74			

See Table 1 for the detail of the type of gastrectomy and postoperative complication

## Discussion

We demonstrated that 13.8% of patients who underwent gastrectomy for GC experienced readmission within 1 year. Choe et al. reported that 11.7% of patients were readmitted within 1 year after gastrectomy [13]. Kim et al. reported that the 5-year readmission rate was 13.0% in patients who underwent radical subtotal gastrectomy for early GC [9], and approximately 80% of these patients were readmitted within 1 year, indicating that the 1-year readmission rate was approximately 10%. The slightly higher 1-year readmission rates in this study compared with the readmission rates in previous results may be due to the inclusion of advanced GC in our study and differences in the health insurance systems.

Many patients who have undergone gastrectomy for GC reportedly experienced nutritional difficulty as gastrectomy reduces the stomach volume. Food intake gradually increases over time in most patients. However, food intake decreases again in some patients after hospital discharge because preparation of suitable food at home is difficult for post-gastrectomy patients. These patients are likely to be rehospitalized for nutritional support. Patients who have undergone gastrectomy for GC are at higher risk of readmission due to poor food intake compared to patients who have undergone surgeries other than gastrectomy. In this study, nutritional difficulty after gastrectomy was identified as the second leading cause of readmission in patients who underwent gastrectomy for GC. In addition, this study revealed that the interval from primary discharge to the first readmission due to nutritional difficulty was significantly shorter than the interval due to other causes. In fact, 50% of readmissions for nutritional difficulty in this study occurred in the first 30 days. Therefore, the short interval from primary discharge to the first readmission is a unique characteristic of readmission due to nutritional difficulty.

Of note, readmission due to nutritional difficulty can be avoided. Baker et al. showed that home enteral nutrition for 6 weeks through a feeding jejunostomy tube did not affect the oral intake of a regular diet and improved postoperative nutrition following TG [6]. Recently, oral nutritional supplements (ONS) have garnered interest as perioperative nutritional interventions in patients with GC who underwent gastrectomy. Kimura et al. demonstrated that administration of 300 kcal/day of ONS for 6 to 8 weeks in the early post-gastrectomy period alleviated weight loss as long as 1 year postoperatively in patients who underwent TG [7]. Although nutritional support may prevent readmission due to nutritional difficulty, providing intensive nutritional support for all patients who underwent gastrectomy for GC is deemed impractical. Therefore, predicting which patients are at high risk of readmission due to nutritional difficulty is necessary to provide intensive nutritional support to these at-risk patients.

In this study, the type of gastrectomy and the mFI were determined to be independent predictive indicators for readmission due to nutritional difficulty. TG and PG were risk factors for readmission due to nutritional difficulty. According to the Post Gastrectomy Syndrome Assessment Study, 1-year bodyweight reduction rates after gastrectomy for GC were 13.8%, 10.9%, 7.9%, and 8.9% in TG, PG, DG with Billroth I reconstruction, and DG with Roux-en-Y reconstruction, respectively [14, 15]. Therefore, patients who underwent either TG or PG are more likely to have nutritional difficulty after surgery than those who have undergone DG. Furukawa et al. recently reported that subtotal gastrectomy with a very small remnant stomach had more favorable short-term outcomes and nutritional status than total and proximal gastrectomy [16]. The small remnant stomach seems to be useful in maintaining ghrelin secretion and reducing gastroesophageal reflux. Preservation of the gastric cardia contributes to a favorable postoperative nutritional status. Therefore, improvements in surgery may reduce readmission due to nutritional difficulty.

Frailty is a syndrome characterized by decreased physiological reserve. It is often associated with an increased risk of adverse outcomes in patients who have undergone surgery [17–20]. A standardized, quantifiable assessment of frailty may enable surgeons to evaluate the risk of adverse outcomes after surgery. Therefore, the development of a useful and less complex tool to evaluate frailty is indispensable for improving patient outcomes. The mFI is one such tool. The mFI is based on the assessment of 11 physiological deficits collected by the American College of Surgeons National Surgical Quality Improvement Program (NSQIP) [11]. These 11 items are easily identifiable during patient encounters and are defined as the proportion of potential deficits that are present in an individual to the 11 potential deficits that were evaluated. The mFI can predict postoperative short-term outcomes in several surgical populations, including patients undergoing abdominal, vascular, and head and neck surgeries [21–24]. However, the correlation between mFI and readmission due to nutritional difficulty is yet to be determined. Choe et al. reported that preoperative assessment of frailty could predict readmission within 1 year of discharge after gastrectomy [13]. In their study, frailty was assessed using the Study of Osteoporotic Fractures Frailty Index. Furthermore, all causes associated with 1-year readmission were included in their study. On the other hand, we used mFI to evaluate frailty, wherein we determined a close correlation between mFI and 1-year readmission due to nutritional difficulty. In our study, frail patients with GC were at high risk of readmission due to nutritional difficulty, indicating that frail patients with GC had more difficulties adjusting to the post-gastrectomy status than adjustment in non-frail patients with GC. To the best of our knowledge, this is the first study to demonstrate the close correlation between mFI and 1-year readmission due to nutritional difficulty.

Our study demonstrated that the combination of mFI and the type of gastrectomy was more useful in predicting readmission due to nutritional difficulty than the use of either of these indicators alone. Because readmission rates due to nutritional difficulty were high in patients who underwent either TG or PG with high mFI, intensive follow-up and nutritional support should be performed in these patients to reduce readmission rates. We recommend that such high-risk patients take ONS in the early post-gastrectomy period, and follow-ups should be conducted every 1 to 2 months to check their nutritional status. Furthermore, education regarding the progression of diet and proper hydration is given to such patients by a nutritionist before discharge and at the outpatient clinic.

This present study had several limitations. First, the retrospective design may be associated with bias. Second, only a small number of patients were included. A larger trial is thus required to confirm our results. Third, all patients included in this study were Japanese. Because insurance systems are different for each country, the indications for readmission might also be different; this is likely to affect the predictive factors for readmission.

In conclusion, we demonstrated that the type of gastrectomy and the mFI were predictive indicators of readmission due to nutritional difficulty in patients who underwent gastrectomy for GC. Because the readmission rate due to nutritional difficulty was high in patients who underwent either TG or PG with a high mFI, intensive follow-up and nutritional support should be provided to these patients to reduce readmission due to nutritional difficulty, improve patient QOL and prognosis, and reduce medical costs.

## Data Availability

The datasets used and/or analyzed during the current study are available from the corresponding author on reasonable request.

## Abbreviations

AUC: Area under curve
DG: Distal partial gastrectomy
GC: Gastric cancer
mFI: Modified frailty index
NSQIP: National Surgical Quality Improvement Program
ONS: Oral nutritional supplements
PG: Proximal partial gastrectomy
PNI: Prognostic nutritional index
ROC: Receiver operating characteristic
TG: Total gastrectomy
USD: United States dollar
